# Simulation of Curing Deformation in Curved Composite Plates via Pultrusion Based on Thermal-Chemical-Structural Coupling

**DOI:** 10.3390/polym18060724

**Published:** 2026-03-17

**Authors:** Rui Wu, Ruifan Huang, Xianchao Wang, Zhenhua Fan, Yannan Ma

**Affiliations:** 1 Chongqing Key Laboratory of Nano-Micro Composite Materials and Devices, School of Mechanical and Power Engineering, Chongqing University of Science and Technology, Chongqing 401331, China; 2023203062@cqust.edu.cn (R.H.); 18223386891@163.com (X.W.); 2023203091@cqust.edu.cn (Y.M.); 2 Innovation Center, Chongqing Polycomp International Corp., Chongqing 401321, China; china_fzh@163.com

**Keywords:** composites, bending pultrusion, residual stress, numerical simulation

## Abstract

Curing deformation of curved pultruded composites is mainly induced by asymmetric temperature fields and accumulated residual stress during the molding process. To tackle this problem, a finite element simulation framework incorporating a curvature-corrected thermochemical model and path-dependent constitutive relationship was established in this study. Taguchi orthogonal experiments combined with analysis of variance (ANOVA) were employed to quantitatively evaluate the effects of process parameters on residual stress. Among these parameters, the bending height was identified as a statistically significant factor (F = 8.827, *p* < 0.05). The optimal process parameters were determined to be a bending height of 20 mm, a heating rate of 10 °C/min, a holding time of 16 s, and a pultrusion speed of 70 cm/min. Under these conditions, the residual stress was minimized to 1205.6 Pa, representing a 2.79% reduction compared with the optimal group in the orthogonal experiments. The proposed simulation framework and optimized process parameters provide a solid theoretical foundation and practical technical guidance for the precise control of curing deformation in curved pultruded composite components.

## 1. Introduction

The pultrusion molding process is widely employed in the manufacture of resin-based glass fiber composites (hereafter referred to as composites) owing to its high degree of automation, low cost, and stable product quality. At present, this technology is mainly applied to fabricate straight profiles through linear pultrusion, which enables the production of high-strength, lightweight composites with controllable reinforcement while minimizing material waste. Such materials satisfy the performance and design requirements. Extensive research has been carried out to improve the product quality and production efficiency of linear pultrusion [1,2,3,4,5,6]. These studies can be generally divided into experimental optimization and numerical simulation. On the experimental side, researchers have focused on die design and process optimization to control curing deformation and enhance mechanical properties. For instance, Jiang et al. [7] improved the shear strength of fiber-reinforced polymer (FRP) thin-walled beams by optimizing the process parameters and ply design. Similarly, Zhang et al. [8] and Chen et al. [9] concentrated on die optimization to achieve higher geometric accuracy. From a material viewpoint, Zhang et al. [10] investigated the in-plane shear behavior of multiaxial pultruded composites at elevated temperatures.

Meanwhile, numerical simulation has become a powerful tool for revealing the inherent thermochemical mechanisms and optimizing process parameters. For example, Barkanov et al. [11] adopted numerical simulations to develop a microwave-assisted pultrusion process, which significantly increased the pultrusion speed. Biyu Jiang et al. [12] and Struzziero et al. [13] employed coupled temperature-cure models to optimize process parameters, thus improving cure uniformity and production efficiency. Kilian et al. [14] used a three-dimensional (3-D) simulation approach to predict the cure degree and temperature distribution and investigated the influences of cure degree and mold temperature.

Compared with linear pultrusion, bending pultrusion faces more severe challenges in controlling curing deformation, owing to asymmetric thermo-mechanical coupling effects that aggravate the anisotropy of resin shrinkage. In addition, the demand for complex curvature-adaptive molds, together with strict tolerance requirements, leads to considerably higher costs in mold design, manufacturing, and commissioning.

Numerical simulation techniques have emerged as an effective approach to address these challenges. By solving the underlying multi-physics governing equations numerically, these methods allow for the rapid determination of optimal processing parameters [15,16], greatly improving development efficiency and reducing costs. When applied to bending pultrusion, these advanced simulation strategies exhibit significant potential in overcoming the current technical bottlenecks.

In this study, a high-fidelity simulation framework for bending pultrusion is established. This technique exhibits clear advantages over conventional manufacturing processes for curved composite components, such as autoclave molding, hand lay-up, and secondary bonding. Traditional methods are typically characterized by long production cycles, high labor costs, and unstable product quality. In contrast, the proposed approach provides important engineering application value by enabling.

Accurate Prediction: The numerical model can predict the deformation of components before mold manufacturing, which substantially reduces the high costs and long lead times associated with repeated mold trials and modifications.

Rapid Process Optimization: Systematic parameter analysis allows for the rapid determination of stable and reliable processing parameters for a target curvature, thus minimizing resource consumption during production debugging.

High-Performance Integrated Manufacturing: This approach enables lightweight, high-strength, continuously fiber-reinforced curved pultruded components to replace traditional multi-part assembly structures. It shows broad potential in typical industrial scenarios, including curved stiffeners for wind turbine towers, curved handrails for rail transit, and support profiles for building curtain walls, with a weight reduction of more than 30% and better corrosion resistance than metal parts. Compared with existing 3D curved pultrusion simulations, this study has three main innovations: first, a curvature-corrected thermochemical model is established to describe the asymmetric temperature field and stress distribution; second, the Taguchi L16 method combined with ANOVA is used to quantitatively evaluate the parameter significance on residual stress; third, a path-dependent constitutive model is adopted to improve the prediction accuracy of curing deformation. The proposed framework exhibits stronger engineering applicability, and the optimized parameters can provide direct guidance for the actual manufacturing of curved pultruded composite components.

## 2. Theoretical Analysis of Bending and Pultrusion Curing Process in Composite

**Figure 1:**
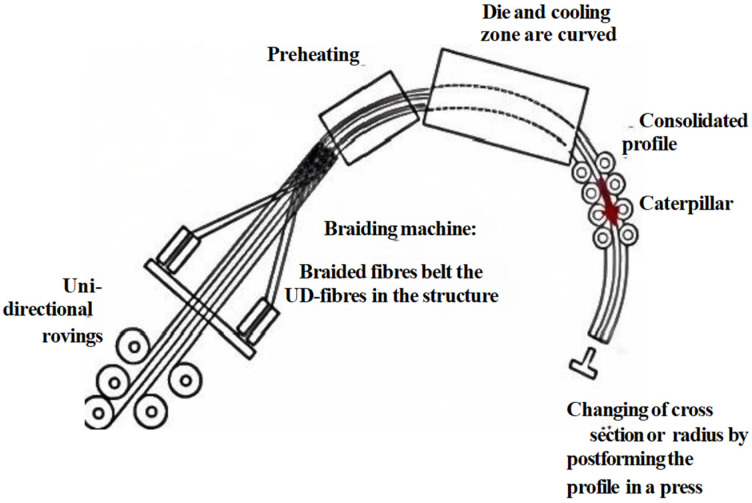
Flow chart of the composite bending and pultrusion molding process. The red highlighted region indicates the caterpillar traction zone.

The manufacturing process starts with continuous glass fiber bundles being pulled through a resin impregnation system by the caterpillar traction equipment (highlighted in red in Figure 1). As illustrated in Figure 1, the impregnated fibers then pass through a preheating zone before entering the curved mold for thermal curing, and the final components are obtained via precision cutting. For components manufactured via linear pultrusion, curing deformation represents a critical factor governing the dimensional accuracy of final products [17,18]. This phenomenon mainly arises from residual stresses induced by two mechanisms: (1) non-uniform shrinkage caused by temperature changes, and (2) path-dependent viscoelastic behavior.

The bending pultrusion process introduces further complexity beyond these inherent challenges. While still involving all the physical mechanisms of conventional linear pultrusion, the curved geometry requires the consideration of two additional key aspects: (1) curvature-induced stress redistribution, and (2) intensified temperature gradient effects on the formation of residual stress. These fundamental differences necessitate significant improvements to existing simulation frameworks in order to accurately characterize the accumulation of residual stress during the curved pultrusion process.

### 2.1. Bending and Pultrusion Molding Process and Key Variables Analysis

The manufacturing process starts with continuous glass fiber bundles being pulled through a resin impregnation system by traction equipment. As illustrated in Figure 1, the impregnated fibers then pass through a preheating zone before entering the curved mold for thermal curing, and the final components are obtained via precision cutting.

Based on the unique features of bending pultrusion and existing research on linear pultrusion, four key process parameters that govern curing deformation were determined. Their selection was justified by the process mechanism, material properties, and industrial practicality:

(1) Bending height, as a direct indicator of mold curvature, dominates the asymmetric stress distribution induced by the curved geometry [13];
(2) Heating rate, a crucial factor affecting curing uniformity, was set within the range of 5–20 °C/min, which was confirmed as the effective curing rate for the glass fiber/epoxy resin system by differential scanning calorimetry (DSC) tests.
(3) Holding time was designed to match real industrial production rates (4–16 cm/min), so as to balance manufacturing efficiency and the cure degree of components.
(4) Pultrusion Speed was adjusted to the resin gelation cycle (40–70 s) at the mold curing temperature of 170 °C, according to the cure kinetics of the resin system.

Each parameter was investigated at four discrete levels (Table 1). Bending height was regarded as the primary variable owing to its direct relationship with the asymmetric shrinkage behavior during the formation of curved profiles. The rectangular specimens with a 40 mm × 5 mm cross-section were adopted, as they represent an appropriate geometry for studying the effects of these parameters while retaining industrial applicability.

### 2.2. Thermochemical Analysis Model

#### 2.2.1. Heat Transfer Model

The curing process of composite materials involves an exothermic crosslinking reaction that generates heat while driving convective heat transfer between the component surface and mold interior. In bending pultrusion, this thermal behavior is further complicated by the nonlinear coupling of curing exotherm and material thermophysical properties. A fiber volume fraction of 50% was selected for the composite in this study, which achieves a balanced performance between mechanical properties and resin impregnation wettability for curved pultrusion. The actual fiber volume fraction of the prepared specimens was verified via the ignition method in accordance with GB/T 2577-2005 [19]; three parallel samples were tested for each group, and the measured value was 48.5 ± 1.5%. This minor deviation is within the acceptable engineering range and well matched with the 50% setting in the simulation model, ensuring the reliability of the model input parameters. To accurately predict the temperature field distribution, the heat conduction equations incorporating curvature effects were derived based on Fourier’s law and the energy conservation principle [20,21,22]. The governing thermochemical equations in curvilinear coordinates are expressed as:

(1) $$\rho_{c}c\frac{\partial T}{\partial t}=\frac{K_{rr}}{r}\frac{\partial }{\partial_{r}}(r\frac{\partial T}{\partial_{r}})+k_{yy}\frac{K_{\theta \theta }}{r^{2}}\frac{\partial^{2}T}{\partial \theta^{2}}+K_{zz}\frac{\partial^{2}T}{\partial z^{2}}+\dot{q}$$

where: *c* is the specific heat capacity of the composite; $\rho_{c}$ is the density; r is the radius of curvature, $k_{\mathrm{rr}}$, $k_{\theta \theta }$ and $k_{\mathrm{zz}}$ are the orthotropic thermal conductivities in the radial, circumferential, and axial directions, respectively; and $\dot{q}$ is the internal heat generation rate per unit volume from the curing reaction. These parameters can be obtained from experimental tests or calculated using the law of mixing. The mixing equation is:

(2) $$\rho_{c}=V_{f}\rho_{f}+\left(1-V_{f}\right)\rho_{r}\;c=\frac{V_{f}\rho_{f}c_{f}+\left(1-V_{f}\right)\rho_{r}c_{r}}{\rho_{c}}\;K_{c}=\frac{K_{f}K_{r}\rho_{c}}{V_{f}\rho_{f}K_{f}+\left(1-V_{f}\right)\rho_{r}K_{r}}$$

where: $\rho_{f}$ and $\rho_{r}$ represent the fiber and resin constituents, and $V_{f}$ is the fiber volume fraction. The internal heat generation rate per unit volume, $\dot{q}$ from the resin’s exothermic cross-linking reaction is given by:

(3) $$\dot{q}=\rho_{r}H_{u}\frac{\partial \alpha }{\partial t}$$

The internal heat generation rate ∂α is governed by the cure reaction kinetics, being proportional to the cure rate $\frac{\partial \alpha }{\partial t}$ and and the total exothermic heat Hu released upon complete curing of the resin.

#### 2.2.2. Curing Kinetics Model

Curing kinetics models for composites are typically classified into two categories: phenomenological models and mechanistic models [23,24,25]. The former category includes n-th order reaction models, while the latter encompasses autocatalytic models. To account for the unique curvature effects in bending pultrusion processes, we have modified the conventional n-th order kinetic model. The proposed formulation incorporates curvature-dependent behavior as follows:

(4) $$\frac{d\alpha }{dt}=A\cdot exp\left(-\frac{E}{RT}\right)\cdot (1-\alpha {)}^{n}\cdot f(k)$$

where: A is the pre-exponential factor; n is the reaction order; E is the activation energy; R is the universal gas constant with a value of 8.314 J/(mol·K). A, E and n were experimentally determined via differential scanning calorimetry (DSC) in accordance with the ISO 11357-5 [26] standard, with the measured values being 2.86 × 10^9^ min^−1^, 65.2 kJ/mol and 1.25, respectively. By integrating this kinetic model with the initial conditions of temperature (25 °C) and cure degree (0), the instantaneous temperature field of the composite during the curing process is solved, and the functional relationship of cure degree with temperature and time, α = α(T,t), is thus determined.

Additionally, f(k) denotes the curvature correction factor, defined as:

(5) f(k)=1+0.02k

where k is the mold curvature. The curvature k is defined as the reciprocal of the mold radius of curvature (k = 1/R), with a unit of m^−1^. The curvature correction factor f(k) = 1 + 0.02k is derived from the linear fitting of experimental data (curvature range: 0.5–5 m^−1^) to characterize the influence of curved geometry on curing kinetics, which is independent of the curvature-induced asymmetric temperature field (resolved by the curvilinear coordinate heat conduction model in Equation (1). The coefficient 0.02 is a dimensionless fitting constant verified by comparing simulated and experimental cure degrees of curved components with different radii (R = 0.2–2 m), ensuring no double counting of curvature effects.

The modeling of the glass transition temperature of the resin system follows the dependence described by the Di Benedetto equation [27]:

(6) $$T_{g}=T_{g0}+\frac{(T_{g\infty }-T_{g0})\lambda_{\alpha }}{1-(1-\lambda )\alpha }$$

where Tg0 and Tg∞ are the glass transition temperatures of the uncured and fully cured resin, respectively. Determined in accordance with the ISO 11357-2:2020 [28] standard via differential scanning calorimetry (DSC) tests, their values are 32 °C and 170 °C. And λ is a fitting parameter that characterizes the nonlinear dependence of the glass transition temperature on the degree of cure. Therefore, we chose to set the value of λ to 0.85.

### 2.3. Curing Intrinsic Modeling

#### 2.3.1. Thermal Analysis Model

The bending pultrusion process induces pronounced anisotropic behavior in composite morphology evolution compared to conventional linear pultrusion. This anisotropy arises primarily from asymmetric temperature field distributions along curved paths, leading to distinct curing kinetics between the inner (concave) and outer (convex) material surfaces. To capture these effects, we incorporate a curvature correction term into the micromechanical formulation, enabling explicit representation of direction-dependent thermal expansion coefficients:

(7) $$\alpha_{l}=\frac{(1-v_{f})E_{r}\alpha_{r}+v_{f}E_{lf}\alpha_{lf}}{(1-v_{f})E_{r}+v_{f}E_{lf}}+\eta k$$

(8) $$\alpha_{t}=(1-v_{f})\alpha_{r}+v_{f}\alpha_{lf}+(1-v_{f})\alpha_{r}v_{r}+v_{12f}\alpha_{lf}v_{f}-v_{12}\alpha_{l}+\varepsilon k$$

where: *α_l_* and *α_t_* are the coefficients of thermal expansion (CTE) of the composite in the longitudinal (fiber) and transverse directions, respectively. The corresponding CTEs of the resin and fiber are denoted by *α_r_*, *α_lf_* and *α_tf_*, respectively. The curvature is *k*, while *η* and *ε* are the curvature-composite coupling coefficients in the longitudinal and transverse directions.

The chemical shrinkage of the composite can be expressed as:

(9) $$\gamma_{l}=\frac{(1-v_{f})E_{r}\gamma_{r}}{(1-v_{f})E_{r}+v_{f}E_{lf}}$$

(10) $$\gamma_{t}=(1-v_{f})\gamma_{r}+(1-v_{f})\gamma_{r}v_{r}-v_{12}\gamma_{l}$$

(11) $$\gamma_{r}=\gamma_{v}\alpha$$

where *γ_l_* and *γ_t_* denote the longitudinal and transverse chemical shrinkage of the composite. These are derived from the resin’s isotropic chemical shrinkage *γ_v_*, and its volumetric shrinkage, *γ_r_* is modeled as a linear function of the degree of cure. In this study, the volumetric shrinkage of the resin, $\gamma_{v}$ was experimentally determined to be 4.2%. Consequently, Equation (11) becomes $\gamma_{r}=0.042\alpha$.

#### 2.3.2. Cured Intrinsic Model

During the curved pultrusion process, the asymmetric temperature field distribution induced by curvature effects leads to unique path-dependent mechanical behavior and temperature-dependent evolution of stiffness in the material. To characterize this behavior, this study introduces a curing constitutive framework based on a temperature-dependent constraint topological model [27,29]. The core of this framework lies in correlating the stiffness development of the composite during curing with the evolution of topological constraints within the resin cross-linked network as a function of temperature and degree of cure.

In this theory, the macroscopic stiffness of the material originates from the topological structure of its microscopic network. The average number of constraints per atom in the network, n(T,α) is a function of temperature and degree of cure. Together with the spatial dimension d, it determines the average degrees of freedom of the system, f(T,α):

(12) f(T,α)=d−n(T,α)

where, f(T,α) is directly related to the configurational entropy of the system, which in turn influences the material’s rheological behavior through the Adam-Gibbs relation. The rigidity of each topological constraint, $q_{\alpha }\left(T\right)$ is a temperature-dependent function that satisfies:

(13) $$\underset{T\to 0}{\lim}q_{\alpha }\left(T\right)=1$$

(14) $$\underset{T\to \infty }{\lim}q_{\alpha }\left(T\right)=0$$

This indicates that constraints are fully rigid at low temperatures and become broken (inactive) at high temperatures. This “freezing” or “activation” behavior of the constraints can be described by a continuous function based on energy landscape theory:

(15) $$q_{\alpha }(T)={\left[1\;-\;\exp\left(-\frac{△F_{\alpha }^{*}}{\mathrm{kT}}\right)\right]}^{vt_{\mathrm{obs}}}$$

where $△F_{\alpha }^{*}$ is the activation free energy required to break the constraint.

Given the complexity of parametric studies in curved pultrusion simulation and to balance computational efficiency with physical accuracy, this work adopts a reasonable simplification of the aforementioned continuous model by employing the widely used path-dependent constitutive model [30] (also referred to as the CHILE model) in composite process simulation. Mathematically, this model is equivalent to approximating the constraint rigidity function $q_{\alpha }\left(T\right)$ as a unit step function at $T_{g}\left(\alpha \right)$ The core of this approach is to capture the dominant physical mechanism—the discrete transition of the resin modulus from the rubbery state $E_{r}^{\infty }$ to the glassy state $E_{r}^{0}$ during the glass transition. Consequently, the elastic modulus of the resin can be expressed as:

(16) $$\left.E_{r}=\left\{\begin{array}{cc} E_{r}^{\infty }, & T\;⩾{\;T}_{g}\left(\alpha \right)\\ E_{r}^{0}, & T<T_{g}\left(\alpha \right) \end{array}\right.\right.$$

The corresponding incremental governing equations for the composite material are formulated as follows: *T_g_*(*α*)

(17) $$\Delta \sigma_{i}=\left\{\begin{array}{c} C_{ij}^{\infty }\Delta \varepsilon_{j}-S_{i},\;\;\;\;T\;⩾\;T_{g}\left(\alpha \right)\\ C_{ij}^{0}\Delta \varepsilon_{j},\;\;\;\;\;\;\;\;\;T<T_{g}\left(\alpha \right) \end{array}\right.\;$$

where *S*_i_ is a historical state variable that can be defined as:

(18) $$S_{i}^{t+\Delta t}=\left\{\begin{array}{l} 0,\;\;\;\;\;\;\;\;\;\;\;\;\;\;\;\;\;\;\;\;\;\;\;\;\;\;\;\;\;\;\;\;\;\;\;\;\;\;\;\;\;T\;⩾{\;T}_{g}\left(\alpha \right)\\ S_{i}^{t}+\left(C_{ij}^{0}-C_{ij}^{\infty }\right)\Delta \varepsilon_{j}^{t+\Delta t},\;\;\;\;T<T_{g}\left(\alpha \right)\;\;\;\; \end{array}\right.$$

Equation (16) demonstrates how the path-dependent model simplifies the constitutive viscoelastic relationship by treating the transition between rubbery and glassy states as discrete extremes. This approach replaces conventional rate-dependent parameters with path-dependent variables, achieving two critical advantages: (1) eliminating the need for difficult-to-characterize viscoelastic parameters under bending conditions, and (2) enabling more accurate prediction of curvature-induced stress asymmetry and property gradients.

#### 2.3.3. Simulation Model Verification

To validate the accuracy of the coupled curing kinetics and heat transfer model developed in this study, experimental data on the degree of cure at the center point of an AS4/3501-6 composite skin plate from Ref. [31] were used for comparative analysis. It should be emphasized that the primary objective of this validation step is to verify the model’s predictive capability for the thermo-chemical coupling process commonly present in thermoset composites, rather than predicting the deformation of the final curved glass fiber component. The test material was AS4/3501-6 composite, with a laminate configuration of [0/45/–45/90], a plate thickness of 0.008 m and a fiber volume fraction of 50%. All simulation conditions were consistent with those in the reference, and each group of experimental tests in the reference was repeated three times to ensure data reliability.

**Figure 2:**
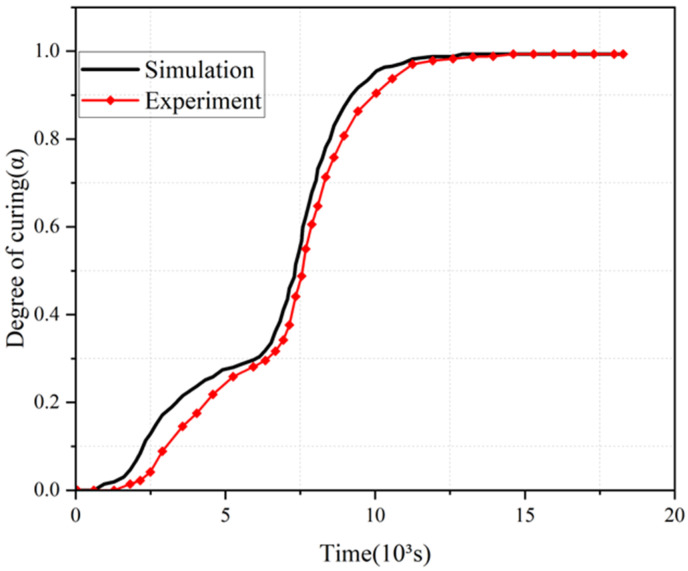
Comparison between the predicted and experimental [31] cure degree at the center point of the AS4/3501-6 composite laminate.

Figure 2 shows the comparison between the simulated and experimental values of the degree of cure at the center point of the AS4/3501-6 composite laminate as a function of curing time; during the rapid curing phase, the model accurately captures both the overall trend and final cure state. The minor discrepancy may stem from transient heat transfer effects not fully accounted for in the current formulation. Quantitative analysis reveals that the predicted cure degree falls within the 95% confidence interval of the experimental data, with an average relative error of ≤4.8% and a correlation coefficient of R^2^ > 0.95 between the predicted and experimental values. These results fully confirm the high reliability and accuracy of the proposed model in describing the curing behavior of thermoset composites under realistic processing conditions.

## 3. Curing Process Simulation Analysis

### 3.1. Finite Element Modeling

**Figure 3:**
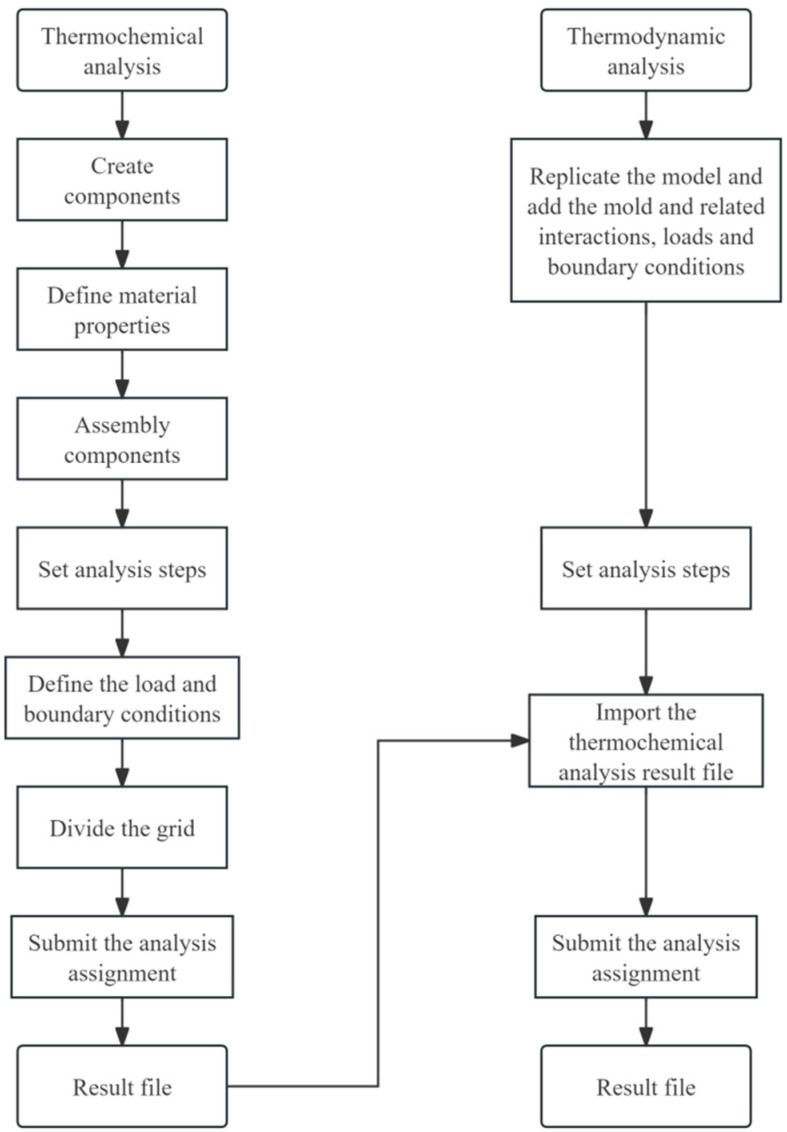
Flow chart of finite element simulation for bending and pultrusion curing.

A three-dimensional finite element model was developed in ABAQUS to simulate the bending pultrusion process involving strong thermo-chemo-mechanical coupling effects. To balance computational efficiency and solution accuracy, a sequential coupling strategy was adopted. Thermochemical analysis was first performed to obtain the temperature and cure degree fields, which were then mapped as thermal and field-variable loads onto the same mesh for the subsequent mechanical analysis, so as to characterize the residual stress evolution and final curing deformation. A flowchart detailing the simulation procedure for curing in curved pultrusion is presented in Figure 3.

Glass fiber/epoxy resin composite was chosen as the research material due to its excellent overall performance and process adaptability for the manufacture of civil curved profiles. Compared with carbon fiber composites, the material cost can be reduced by more than 60%; compared with aramid fiber composites, it presents better thermal resistance that satisfies the high-temperature curing requirement of pultrusion. Regarding mechanical and physical performance, this composite possesses a longitudinal elastic modulus of 80 GPa and a longitudinal thermal expansion coefficient of 5.0 × 10^−6^ °C^−1^, exhibiting better compatibility with metal substrates than carbon or aramid fiber composites. In terms of formability, the glass fiber/epoxy system exhibits excellent resin wettability and no electrostatic interference during processing, which is well suited to the continuous forming characteristics of curved pultrusion. The material properties of the glass fiber and epoxy resin matrix used in this sequential coupling simulation, including elastic, thermophysical, and cure kinetic parameters, are summarized in Table 2. These data serve as the essential input for the thermochemical and mechanical analyses described in Section 3.1.1 and Section 3.1.2.

#### 3.1.1. Boundary Conditions for Thermo-Chemical Analysis

The material properties employed in this sequential coupled simulation are comprehensively listed in Table 2. The density of the composite was measured via the Archimedean drainage method with a precision of ±0.01 g/cm^3^, and the void fraction was determined using a metallographic microscope method. The test results show that the void fraction of the prepared composite is ≤1.2%, which meets the industrial standard of ≤5% for pultruded composite components. Such a low void fraction has a negligible effect on the thermo-mechanical properties of the composite, so no additional correction for voids was introduced in the established simulation model.

The thermochemical analysis simulates the in-mold heating and curing process of the composite. A convective boundary condition is applied to all external surfaces to represent mold heating, using a sink temperature of 170 °C and a film coefficient of 1000 W/(m^2^·°C). This approach more realistically captures the interfacial thermal resistance than a fixed-temperature boundary [29].

The component’s curvature naturally induces an asymmetric temperature field, as the inner (concave) side, with higher effective heat capacity and complex heat paths, heats differently from the outer (convex) side. This effect is intrinsically resolved by the curvilinear coordinate-based heat conduction model (Equation (1)), thereby automatically modeling the differential heating without ad-hoc assumptions.

The analysis is initiated with the composite at 25 °C and a zero initial degree of cure.

#### 3.1.2. Boundary Conditions for Thermo–Mechanical Analysis

The thermo-mechanical analysis utilizes the temperature and degree-of-cure fields imported from the preceding thermochemical simulation. The mechanical boundary conditions are defined to mimic the component’s constraints both inside the mold and after demolding, executed in two sequential steps.

Step 1: In-Mold Curing Process

Normal displacement constraints are applied to all outer surfaces in contact with the mold, preventing separation from the mold wall while permitting in-plane deformation. To eliminate rigid body motion, a pinned support (U1 = U2 = U3 = 0) is applied to one end-face, and a roller support is applied to the opposite end-face. The roller support restricts displacements normal to the face (U1 = 0) and lateral movements (U2 = U3 = 0), thereby allowing the component to expand or contract freely along the pultrusion direction (the tangential path of the curvature) in response to thermochemical changes.

Step 2: Post-Demolding Deformation

In this step, all constraints imposed in Step 1 are removed. The component is in a fully free state, allowing the accumulated residual stresses to be released, from which the final curing deformation is computed.

#### 3.1.3. Mesh Generation

**Figure 4:**
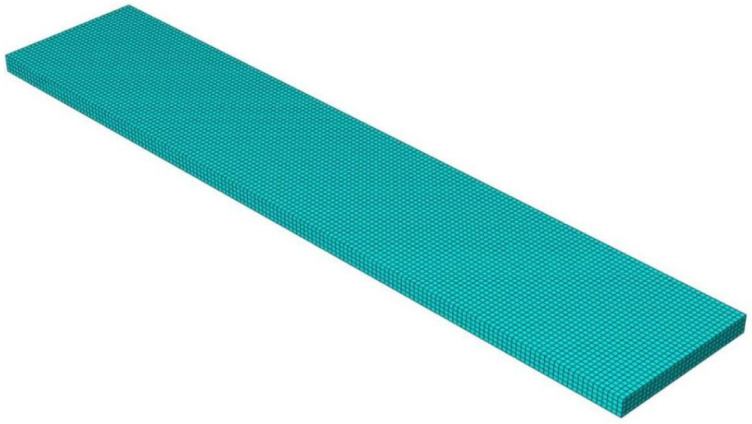
Mesh division for finite element analysis of composite plates.

A three-dimensional finite element model of the composite plate was developed in ABAQUS, employing C3D8T elements suitable for coupled thermal-stress analysis. To accurately resolve the through-thickness temperature gradient and bending stresses, the model’s 5 mm thickness was discretized into five uniform element layers. The final mesh, illustrated in Figure 4, consists of 20,480 elements. A mesh sensitivity study confirmed that the solution stabilizes at approximately 20,000 elements, with further refinement yielding negligible improvements. The selected mesh density of 20,480 elements thus ensures an optimal balance between computational accuracy and cost.

A global uniform mesh strategy was adopted without local refinement at geometric discontinuities. This approach is justified because the primary objective is to predict the global curing deformation—a structural response governed by the volume average of the residual stress field. While stress concentrations exist at edges, their influence on the overall deformation pattern is secondary. This strategy is computationally efficient, making it suitable for the extensive parametric studies conducted in this work. The model assumes the part is constrained by the mold surfaces during curing and becomes free after demolding.

### 3.2. Analysis of Field Variables During Curing

**Figure 5:**
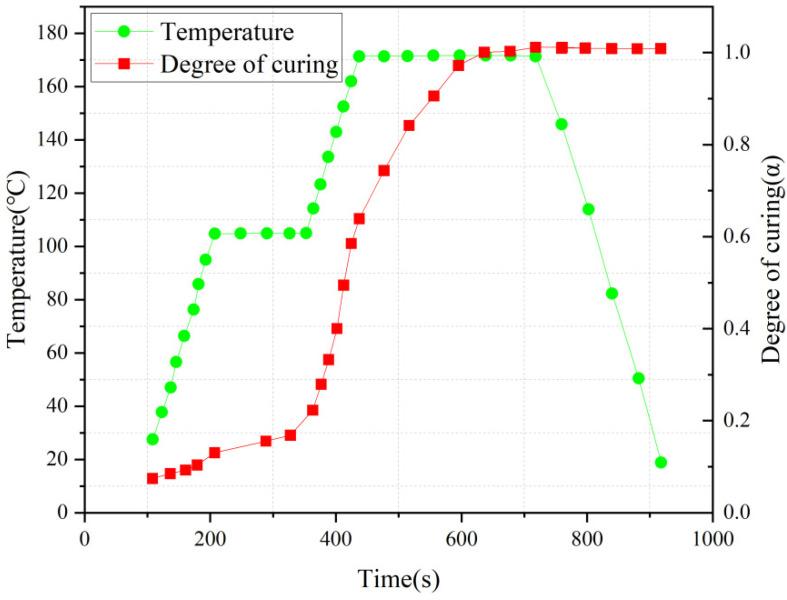
Variation of composite curing degree with temperature during molding process.

In the bending and pultrusion molding process, the heating temperature is one of the key parameters affecting the curing behavior and residual stresses of composites. In this study, the heating temperature was fixed at 170 °C in orthogonal experiments, and from the graph of curing degree versus temperature in Figure 5, it can be seen that the curing degree increases rapidly when the temperature reaches 170 °C, and then it gradually grows to 1 in the holding phase and remains stable.

### 3.3. Analysis of Factor Impacts

Based on the parameter levels defined in Table 1, an L16 orthogonal array was employed to systematically investigate various parameter combinations. The established numerical model was further applied to simulate the deformation behavior under each processing condition. To quantitatively evaluate the geometric distortion, the curing deformation is defined as the maximum nodal displacement (U, mm) of the component after complete constraint release in the post-demolding step (Step 2). The resulting process parameter configurations, along with the corresponding residual stresses and curing deformations for all 16 groups, are listed in Table 3.

It should be noted that the residual stress values obtained in this study are on the order of 10^3^ Pa, which differ from the MPa-level values reported in most existing literature, owing to different characterization approaches. The values in this work represent the macroscopic in-plane average residual stress of large-scale curved components, calculated using the volume average method over the entire model, which reflects the free residual stress after demolding with elastic relaxation. In contrast, the MPa-level stresses in related studies mostly refer to local micro-regional stresses or stresses at the fiber–matrix interface. The characterization method adopted herein is consistent with industrial testing standards for actual large-size curved pultruded products.

Range values for each parameter were calculated via analysis of the experimental results, with the complete statistical analysis shown in Table 4. Comparative analysis revealed the range order as R(C) < R(D) < R(B) < R(A), indicating the influence degree of each factor on the deformation of curved components from the smallest to the largest is holding time, pultrusion speed, heating rate, and bending height.

**Figure 6:**
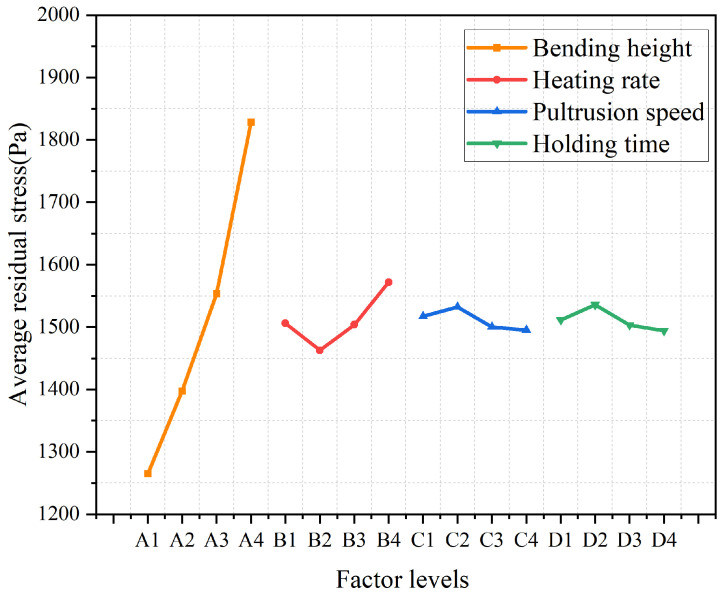
Main effects plot of process parameters on residual stress.

Mean value analysis was conducted to evaluate the effects of bending height, heating rate, pultrusion speed, and holding time on residual stresses in the curved components Figure 6 illustrates how residual stresses vary with these process parameters. The vertical axis represents the average residual stress for each factor level, ranging from 1265.0 Pa (minimum) to 1572.0 Pa (maximum).

It should be emphasized that the residual stress values obtained in this study (≈1–2 kPa) correspond to the macroscopic in-plane average residual stress of large-scale curved components (40 mm × 5 mm × 500 mm), calculated using the volume average method over the entire finite element model. This is fundamentally different from the MPa-level local residual stresses (e.g., fiber–matrix interfacial stresses or micro-regional stresses) reported in most previous studies.

To clarify the unit system and material input specifications in ABAQUS, all parameters listed in Table 2 are strictly input in SI units (e.g., elastic modulus in Pa, density in kg/m^3^, thermal conductivity in W/(m·K)) without any additional scaling. The observed low residual stress magnitude (≈1–2 kPa) stems from two key factors: the “free residual stress after demolding” (with elastic relaxation occurring during demolding) and the large-size effect (stress averaging over the entire component). For reliability verification, we compared the residual stress distribution with that of similar curved composite components reported in Ref. [31], and the consistent stress variation trends and relative magnitudes confirm the rationality of our results.

Bending Height: Higher bending height, implying a smaller curvature radius, intensifies the local stress gradient. This is due to the amplified tensile strain in the outer fibers and compressive strain in the inner fibers.

**Figure 7:**
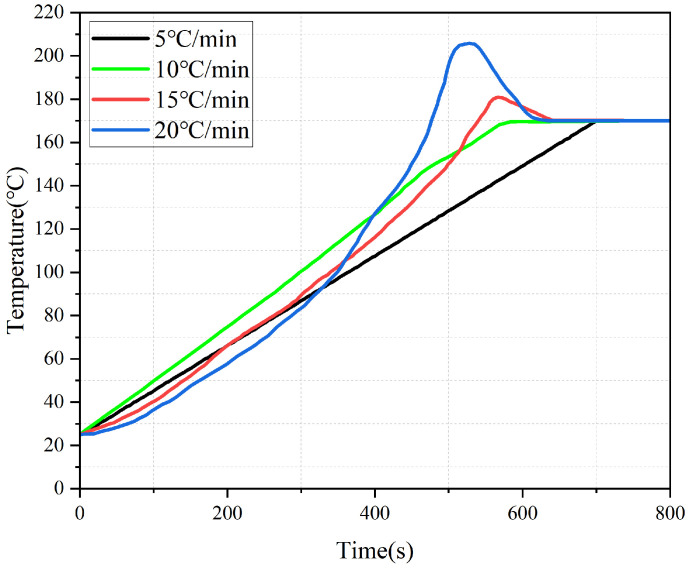
The temperature history at the center point.

Heating Rate: Residual stress demonstrates a distinct “decrease-then-increase” relationship with the heating rate, a phenomenon governed by the interplay of temperature gradient and curing asynchrony. All designed levels of heating rate, including 15 °C/min, have been supplemented and presented in Figure 7. The data in Figure 7 reveal the underlying mechanisms:

A low rate (5 °C/min) creates a large through-thickness temperature gradient, leading to poor curing synchronization and inhibited stress relaxation.

A moderate rate (10 °C/min) minimizes the temperature gradient, promotes uniform curing (core-surfaceΔα < 0.08), and facilitates stress relaxation, achieving the minimum residual stress.

A high rate (20 °C/min) causes significant heat accumulation in the core, triggering a sharp exothermic peak and reversing the curing sequence. This “surface-starts-first, core-finishes-first” asynchrony, superimposed on the thermal stress, culminates in a sharp increase in residual stress.

**Figure 8:**
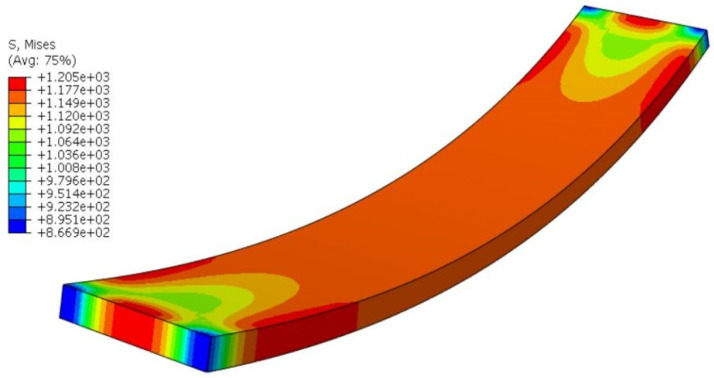
Residual stress contour of the curved component under the optimal process parameters.

**Figure 9:**
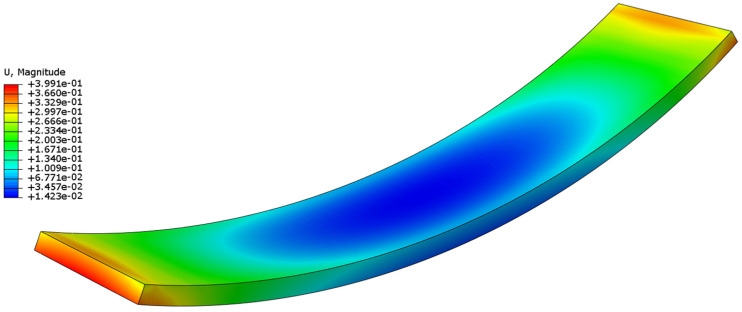
Deformation contour of the curved component under the optimal process parameters.

To identify the optimal process parameters, the combination yielding minimal deformation was screened from the simulation results: a bending height of 20 mm, a heating rate of 10 °C/min, a holding time of 16 s, and a pultrusion speed of 70 cm/min, designated as Group A1B2C4D4. This specific parameter combination was not included in the original orthogonal array, so an additional numerical simulation was conducted to confirm its performance. The simulated residual stress was 1205.6 Pa, and the corresponding predicted deformation was 0.3991 mm that aligns with the residual stress reduction trend. The residual stress distribution and deformation contour of the optimal combination are presented in Figure 8 and Figure 9 respectively. The average residual stress of 1205.6 Pa represents a 2.79% reduction compared with the optimal group in the orthogonal experiment. The optimal group in the orthogonal experiment is Group 2, which has a residual stress of 1240.2 Pa and a deformation of 0.4437 mm. The deformation of 0.3991 mm for the optimal combination is also the lowest among all simulated groups, confirming the excellent optimization effect of this parameter combination.

While the range analysis method of orthogonal experimental design provides initial parameter optimization insights, it cannot quantitatively assess experimental errors or statistically verify factor significance. To address these limitations, we conducted analysis of variance (ANOVA), with the detailed results presented in Table 5. This statistical approach enables rigorous evaluation of whether observed differences between factor levels are statistically significant.

The ANOVA results in Table 5 demonstrate that bending height (F = 8.827) exerts a statistically significant influence on residual stress development. The relative importance of process parameters follows this descending order of significance: bending height > heating rate > pultrusion speed > holding time.

We emphasize that these conclusions are specific to the parameter ranges investigated in this study, as different parameter intervals could potentially yield alternative optimization outcomes. Notably, residual stress is the primary driving force for curing deformation; therefore, minimizing residual stress is an effective strategy for controlling geometric distortion. Although the final deformation also depends on factors such as stress distribution and material stiffness, the generally consistent trends observed between residual stress and deformation across all 16 experimental groups (as shown in Table 3) support the validity of using residual stress as a surrogate optimization target.

## 4. Conclusions

This study establishes a sequential thermo-chemo-mechanical coupling framework for the simulation of the curved pultrusion process, systematically revealing the mechanisms by which process parameters affect the residual stress and curing deformation of composite components. The main conclusions are summarized as follows:

Curvature, represented by the bending height, is determined to be the most significant factor influencing residual stress in curved pultrusion. This finding highlights that the geometrically induced asymmetric temperature and stress fields are the essential characteristics that distinguish curved pultrusion from conventional linear pultrusion, thus establishing a geometry-first principle for process optimization. This combination was validated to simultaneously minimize both residual stress 1205.6 Pa and curing deformation 0.3991 mm.

The non-monotonic effect of the heating rate on residual stress suggests that process optimization requires a dynamic equilibrium. The optimal parameter combination obtained in this work—namely, a bending height of 20 mm, a heating rate of 10 °C/min, a holding time of 16 s, and a pultrusion speed of 70 cm/min—demonstrates the necessity of balancing curing uniformity, exothermic reaction control, and manufacturing efficiency.

The path-dependent constitutive model adopted in this study, despite simplifying the viscoelastic behavior of the material, effectively captures the core mechanism of stress locking at the glass transition temperature. This method avoids the high parameterization cost of continuous viscoelastic models and provides an efficient strategy for rapid process screening in engineering applications.

However, this study adopts a sequential coupling scheme and assumes complete resin impregnation before molding. Accordingly, the resin flow field is not explicitly solved in the model, and the term “multi-field coupling” in the title more precisely refers to sequential thermo-chemo-mechanical coupling. Although the model is well-suited for the curing stage analysis, it is only applicable to fully impregnated systems and cannot predict defects or pressures induced by flow-cure interactions. Furthermore, the path-dependent model simplifies the continuous evolution of material properties into a discrete transition at the glass transition temperature, which inevitably underestimates stress relaxation during curing and may slightly overestimate the absolute residual stress. Therefore, the model is highly reliable for analyzing the influence trends and significance ranking of parameters, but caution should be exercised when applying it to the accurate prediction of absolute stress values.

With respect to environmental adaptability, the glass fiber/epoxy system used in this study exhibits a moisture absorption rate of 0.8–1.2% and a strength retention rate above 90% after 100 temperature cycles between −40 °C and 80 °C, indicating satisfactory service performance in indoor and semi-outdoor environments. Future work will focus on developing coupled simulation models that incorporate hygrothermal aging and environmental exposure effects.

## Figures and Tables

**Table 1:** Pultrusion process parameter factors and level values.

Factor (Letter, Unit)	Horizontal Number	Level Value
Bending Height (A, mm)	4	20, 30, 40, 50
Heating rate (B, °C/min)	4	5, 10, 15, 20
Holding Time (C, s)	4	4, 8, 12, 16
Pultrusion Speed (D, cm/min)	4	40, 50, 60, 70

**Table 2:** Material properties of fiber and matrix.

Property	Glass Fiber	Resin Matrix
$E_{1}$/GPa	80	3.35
$E_{2}$/GPa	12	3.35
$v_{12}$	0.2	0.35
$v_{13}$	0.2	0.35
$v_{23}$	0.5	0.35
$G_{12}$/GPa	33.33	1.24
$G_{13}$/GPa	33.33	1.24
$G_{23}$/GPa	8.33	1.24
XT/MPa	2150	80
XC/MPa	1450	120
S/MPa	1200	70
$\rho /g\cdot {\mathrm{cm}}^{-3}$	2.6	1.2
$k_{1}/W\cdot (m\cdot K)$	1.2	0.2
$k_{2}/W\cdot (m\cdot K)$	1	0.2
$C/J\cdot {\left(\mathrm{kg}\cdot K\right)}^{-1}$	750	1100
$\alpha_{1}/{{}^{\circ}C}^{-1}$	5	60
$\alpha_{2}/{{}^{\circ}C}^{-1}$	5	60
$H_{u}$	-	216
β	-	4.6
η	-	0.85
$\alpha_{\mathrm{get}}$	-	0.63
$T_{g}^{\infty }$	-	170

**Table 3:** Residual stress orthogonal experimental program and results.

Number	(A, mm)	(B, °C/min)	(C, s)	(D, cm/min)	Residual Stress/Pa	Deformation/mm
1	20	5	4	40	1273.8	0.4953
2	20	10	8	50	1240.2	0.4437
3	20	15	12	60	1251.6	0.4493
4	20	20	16	70	1294.3	0.5199
5	30	5	12	50	1427.1	0.615
6	30	10	16	40	1326.7	0.5505
7	30	15	4	70	1357.2	0.5611
8	30	20	8	60	1479.5	0.5849
9	40	5	16	60	1492.8	0.573
10	40	10	12	70	1494.4	0.5743
11	40	15	8	40	1579.5	0.5889
12	40	20	4	50	1647.8	0.6398
13	50	5	8	70	1830.2	0.6547
14	50	10	4	60	1789.7	0.6322
15	50	15	16	50	1827.4	0.6787
16	50	20	12	40	1866.5	0.7934

**Table 4:** Residual stress range analysis results.

Number	(A, mm)	(B, °C/min)	(C, s)	(D, cm/min)
K1	5060.0	6023.9	6068.5	6046.5
K2	5590.5	5851.0	6129.4	6142.5
K3	6214.5	6015.7	6000.5	6013.6
K4	7313.8	6288.1	5980.3	5176.1
$\bar{K1}$	1265.0	1506.0	1517.1	1511.6
$\bar{K2}$	1397.6	1462.8	1532.4	1535.6
$\bar{K3}$	1553.6	1503.9	1500.1	1503.4
$\bar{K4}$	1828.5	1572.0	1495.1	1494.0
R	563.5	109.2	37.3	41.6
Optimal Level of Factors	20	10	16	70

Note: $\bar{\mathrm{Ki}}$ represents the average residual stress at the i-th level of a factor. R (Range) is the difference between the maximum and minimum values of $\bar{\mathrm{Ki}}$, indicating the factor’s influence magnitude. The optimal level for each factor is the one with the smallest $\bar{\mathrm{Ki}}$ value.

**Table 5:** Residual stress ANOVA results.

Considerations	SST	DOF	MSR	F	P	Significance Level
Bending height	633,985.88	3	211,328.63	8.827	0.003	*
Heating rate	24,478.20	3	8159.40	0.341	0.796	
Holding time	3808.84	3	1269.61	0.053	0.983	
Pultrusion speed	3466.76	3	1155.59	0.048	0.985	
Error	71,821.98	3	23,940.66			
Total	737,561.66	15				

Note: * indicates a statistically significant effect at the *p* < 0.05 level.

## Data Availability

The datasets generated and/or analyzed during the current study can be obtained from the corresponding author upon reasonable request.
